# Detection of *MET* Gene Copy Number in Cancer Samples Using the Droplet Digital PCR Method

**DOI:** 10.1371/journal.pone.0146784

**Published:** 2016-01-14

**Authors:** Yanni Zhang, En-Tzu Tang, Zhiqiang Du

**Affiliations:** Amgen Biopharmaceutical Research & Development (Shanghai) Co., Ltd, Shanghai, China; University of Connecticut Health Center, UNITED STATES

## Abstract

**Purpose:**

The analysis of *MET* gene copy number (CN) has been considered to be a potential biomarker to predict the response to MET-targeted therapies in various cancers. However, the current standard methods to determine *MET* CN are SNP 6.0 in the genomic DNA of cancer cell lines and fluorescence *in situ* hybridization (FISH) in tumor models, respectively, which are costly and require advanced technical skills and result in relatively subjective judgments. Therefore, we employed a novel method, droplet digital PCR (ddPCR), to determine the *MET* gene copy number with high accuracy and precision.

**Methods:**

The genomic DNA of cancer cell lines or tumor models were tested and compared with the *MET* gene CN and *MET/CEN-7* ratio determined by SNP 6.0 and FISH, respectively.

**Results:**

In cell lines, the linear association of the *MET* CN detected by ddPCR and SNP 6.0 is strong (Pearson correlation = 0.867). In tumor models, the *MET* CN detected by ddPCR was significantly different between the *MET* gene amplification and non-amplification groups according to FISH (mean: 15.4 vs 2.1; *P* = 0.044). Given that *MET* gene amplification is defined as *MET* CN >5.5 by ddPCR, the concordance rate between ddPCR and FISH was 98.0%, and Cohen's kappa coefficient was 0.760 (95% CI, 0.498–1.000; *P* <0.001).

**Conclusions:**

The results demonstrated that the ddPCR method has the potential to quantify the *MET* gene copy number with high precision and accuracy as compared with the results from SNP 6.0 and FISH in cancer cell lines and tumor samples, respectively.

## Introduction

In normal physiological functions, MET is expressed in cells of epithelial origin, where it plays an essential role in cell growth and homeostasis [1]. However, aberrant MET signaling has been observed in multiple human cancers, including hepatic and gastric cancers [2–6]. *MET* gene amplification has been reported to be correlated with poor prognosis in patients with GC [7–9] and may be used as a potential biomarker to estimate the disease prognosis or predictive response to MET inhibitors in clinical trials. Thus, it is important to develop an accurate and optimized platform to determine the *MET* gene copy number prior to MET-targeted therapy. In routine studies, SNP 6.0 and FISH are the standard methods to evaluate the *MET* copy number of cancer cell lines *in vitro* and tumor samples *in vivo*, respectively. However, these methods have many limitations, including the advanced technical skills required high costs and the need for experienced experts to analyze the tumor samples, which results in variable results between labs. For example, FISH analysis is usually performed on formalin-fixed, paraffin-embedded (FFPE) tissues, which requires a number of complex processes, such as fixation and immunohistological staining, and may cause genomic DNA damage and fracture. These issues increase the probability of false negative results because of the low quality and quantity of DNA. Therefore, the detection of gene amplifications is challenging because of the low sensitivity of these approaches.

The droplet digital PCR (ddPCR) is a method that can absolutely quantify the *MET* copy number without need for standard curves. In a typical digital PCR, the sample is randomly distributed into discrete partitions such that some contain no nucleic acid template and others contain one or more template copies. The partitions are PCR amplified to end point and then read using a droplet reader to determine the fraction of positive partitions based on fluorescence amplitude, from which the absolute concentration of the target or reference DNA is estimated statistically by modeling as a Poisson distribution. Therefore, ddPCR is an end-point measurement that enables to quantify nucleic acids without the need for standard curves, external calibrators and endogenous controls [10].

In this study, we sought to employ ddPCR assays to absolutely quantify the *MET* copy number in cancer cell lines and tumor samples and compared our results with those obtained using SNP 6.0 and FISH for the same samples.

## Materials and Methods

### Cell lines, PDX samples, and extraction of genomic DNA

Eight human GC and thirty-eight HCC cell lines were purchased from five organizations: the American Type Culture Collection (ATCC), Japanese Collection of Research Bioresources (JCRB), Korean Cell Line Bank (KCLB), Shanghai Institutes of Biological Sciences, CAS (SIBS), and Zhongshan Hospital Fudan University (ZHFU) (see Table 1). The cell lines were routinely cultured in 96-well plates in ATCC’s recommended growth medium at 37°C, 5% CO_2_ and 95% humidity. Genomic DNA was extracted from the cancer cell lines with the TIANampGenomic DNA Kit (Tiangen, Cat: DP304) according to the manufacturer’s instructions. One hundred and fifty-six FFPE of patient-derived xenograft (PDX) models (including 116 GC and 39 HCC models) were obtained from the Shanghai LIDE Biotech Company. For the PDX model samples, genomic DNA was extracted using the DNeasy Blood and Tissue Kit (Qiagen, Cat#: 69509) according to the manufacturer’s protocol. The DNA concentration was determined with a NanoDrop spectrophotometer (Thermo), and digested with the HaeⅢ enzyme (NEB, Cat#: R0108S) at 37°C for 1 h. The digested DNA sample was diluted 10-fold with autoclaved Millipore H_2_O and stored in a -20°C freezer.

**Table 1:** Comparison of *MET* gene amplification measured by ddPCR versus FISH in gDNA of FFPE tissue.

gDNA of FFPE samples	ddPCR		
FISH	N	P	Total
**N**	147	3	150
**P**	0	5	5
**Total**	147	8	155

### DDPCR for the determination of *MET* CN

The TaqMan PCR reaction mixture was assembled in a final volume of 20 μL with 2x Supermix, 20x primers and 20x probes and 20 ng of the genomic DNA as the template. Each reaction mixture was then loaded into a DG8 cartridge (Bio-Rad) with 70 μL of droplet generation oil to generate a droplet. The droplets from each well were then transferred into a 96-well PCR plate. The plates were heat-sealed and then thermally cycled under the following conditions: 95°C for 10 min (one cycle); 40 PCR cycles of 95°C for 15 seconds and 60°C for 1 min; and a hold at 4°C. After PCR, the plates were placed on a QX200 droplet reader (Bio-Rad) that analyzed the droplets of each well of the plate and quantified the target DNA. The PCR data were analyzed using QuantaSoft version 1.7.4.0917 (Bio-Rad) to determine the copy number variation (CNV). The 20x reference assay for AP3B1 included primers targeting the centromere loci on Chromosome 5 and a probe labeled with a HEX fluorescent signal (Bio-Rad). The 20 x CNV PCR assay for MET included primers targeting the region from intron 20-exon 21 on Chromosome 7, and the probe was labeled with a FAM fluorescent signal (Life Technologies, cat #Hs02884964_cn). Each well was replicated for all of the samples. The *MET* copy number was calculated as the ratio of the concentrations of *MET* and *AP3B1* and multiplied by two. Each data for *MET* CN using ddPCR represents two or three merged technical replicate wells with no template control (NTC) as a negative control.

### SNP 6.0 assay

Among the 46 cell lines, the SNP 6.0 raw data for 35 of the cell lines were downloaded from the CCLE project (http://www.broadinstitute.org/ccle/home), and the raw data for the other 11 cell lines were collected using the Affymetrix Genome-Wide Human SNP Array 6.0 platform, including BEL7402, HCCC-810, NOZ, OCUG-1, OZ, QGY7701, QGY7703, SMMC7721, SNU354, SNU368, and SNU739. All of the raw data were processed using PICNIC software and presented as *MET* copies.

### *MET* FISH

*MET* gene amplification was analyzed by FISH using the Dako *MET/CEN-7* IQISH Probe Mix (RUO). The *CEN-7* centromere probe was used as a reference control, according to the manufacturer’s instructions. The formalin-fixed, paraffin-embedded specimens (cell pellets) were sectioned and then subjected to deparaffinization and rehydration. H&E staining was performed first. The heat pretreatment was performed in pretreatment solution in a microwave oven for 3 min and 50 sec, cooled to RT over 15 min, and then washed. The sections were digested in RTU pepsin for 6 min at 37°C (the time can be adjusted for different section thicknesses). After washing, the sections were completely dehydrated. The sections containing the probe were denatured at 66°C for 10 min and then hybridized at 45°C for 1–2 h. After stringent washing, the sections were dehydrated, mounted in medium with DAPI, and stored in the dark at 4°C for 15 min before reading. A fluorescence microscope and appropriate filters were used to scan and identify the relevant tumor area. The signals of the red MET gene and green CEN-7 gene were counted in 20 tumor nuclei in a minimum of two areas to determine the *MET/CEN-7* ratio. The FISH scoring was defined as follows: ratio ≤2.0: *MET* amplification was not observed; ratio >2.0: *MET* amplification was observed. If the ratio was borderline (1.8–2.2), an additional 20 nuclei were counted, and the ratios were recalculated.

### Statistical analysis

The valid HCC and GC measurements for *MET* CN were combined for the statistical analyses. Pearson’s correlations and linear regression were used to evaluate the relationship between ddPCR and SNP 6.0 or FISH. To compare the differences in *MET* CN analyzed by ddPCR between the gene amplification and non-amplification groups analyzed by FISH, *t*-tests were used. The consistency of gene amplification based on ddPCR and FISH was evaluated according to the concordance rate and Cohen's kappa coefficient. The analyses were performed with SAS 9.4 software.

## Results

### Comparison of the *MET* CN test between ddPCR and SNP 6.0 in cancer cell lines

**Fig 1:**
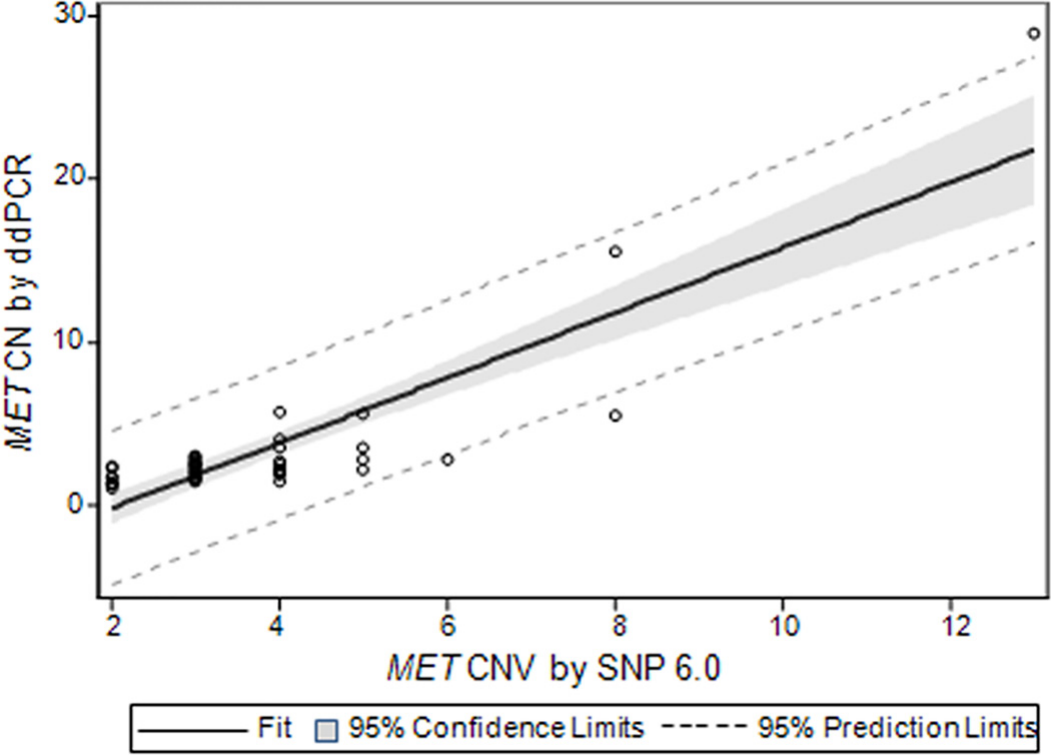
Measurement of *MET* copy number in cancer cell lines using ddPCR versus SNP 6.0. The data is based on a linear regression model that adjusts for SNP 6.0 with intercept. Solid line indicates fitting curve; gray box represents 95% confidence limits; dashed line depicts 95% prediction limits. Each data for *MET* CN using ddPCR represents two or three merged technical replicate wells with no template control (NTC) as a negative control.

Genomic DNA was obtained from 8 GC and 38 HCC cell lines for *MET* copy number detection, and the results of the ddPCR assay were compared to those of SNP 6.0 to determine whether ddPCR can replace the standard molecular biology techniques. The results were shown in S1 Table. We determined the *MET* copy numbers of cell lines *in vitro* using an Affymetrix microarray. Next, we used ddPCR to test the *MET* copy number in the same samples by normalizing to the *AP3B2* reference gene. We observed that the cell lines with high *MET* copy numbers according to SNP 6.0 also had high ddPCR measurements. The linear association for *MET* copy number measurements between ddPCR and SNP 6.0 is strong based on Pearson’s correlation (r = 0.867; *P* <0.001). Moreover, the slope and intercept of *MET* CN by SNP 6.0 to ddPCR in the linear regression were significant (*P* <0.001) with a value equal to 1.996 (standard errors of estimates = 0.177) and -4.159 (standard errors of estimates = 0.752), respectively (Fig 1).

### *MET* gene amplification analysis in GC and HCC PDX models

**Fig 2:**
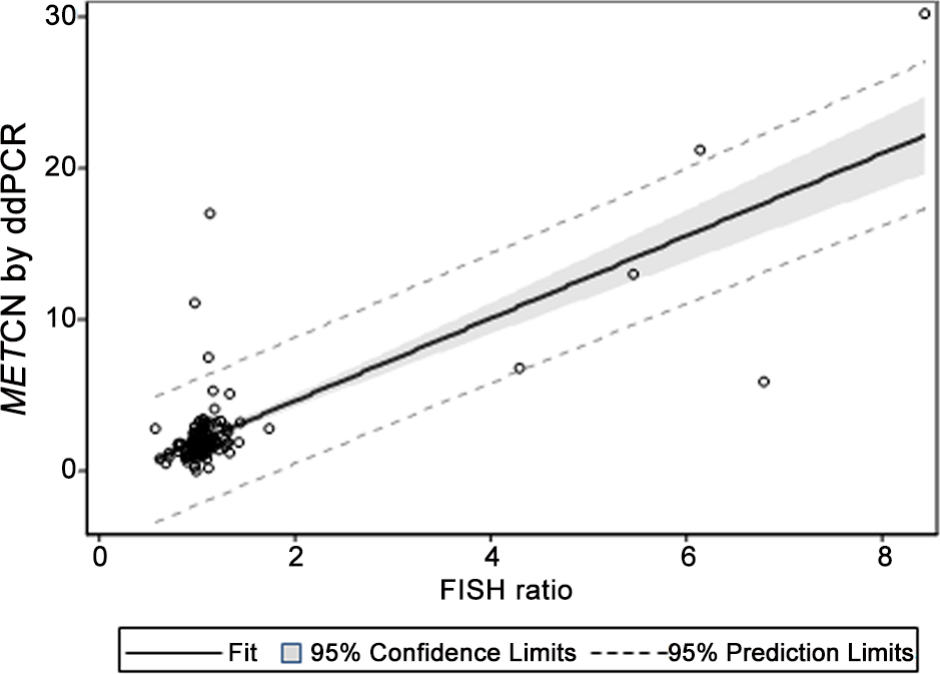
Distribution of *MET* CN measured by ddPCR in *MET* amplification and non-amplification groups by FISH. *P*-value was based on *t*-test assuming difference variances of each group. Each data for *MET* CN using ddPCR represents two or three merged technical replicate wells with no template control (NTC) as a negative control.

**Fig 3:**
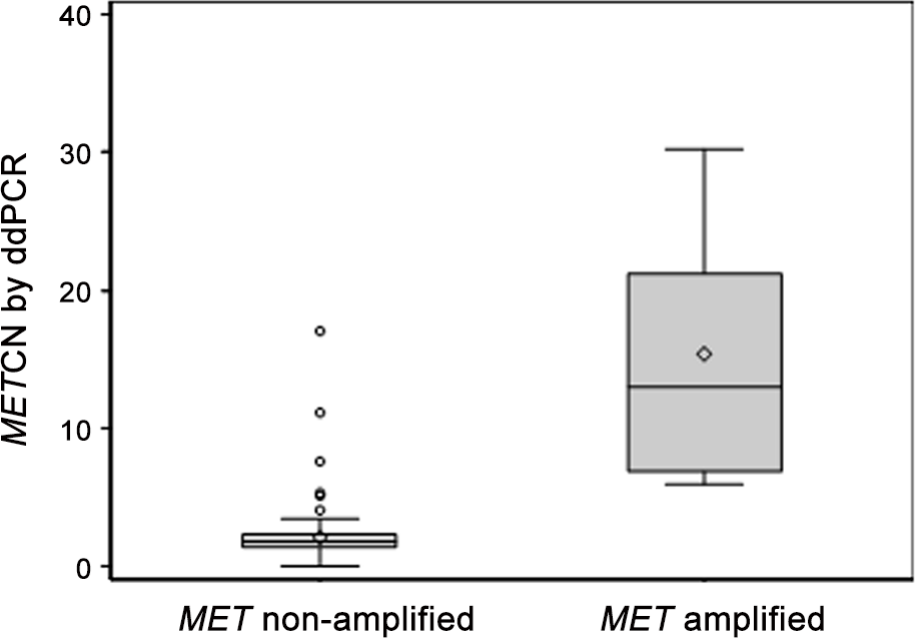
Measurement of *MET* copy number in PDX models using ddPCR versus FISH. The data is based on a linear regression model that adjusts for FISH ratio with intercept. Solid line indicates fitting curve; gray box represents 95% confidence limits; dashed line depicts 95% prediction limits. Each data for *MET* CN using ddPCR represents two or three merged technical replicate wells with no template control (NTC) as a negative control.

Because ddPCR could accurately evaluate the *MET* copy number in cancer cell lines, we then determined whether ddPCR could detect the presence of *MET* amplification and compared the results to those obtained from FISH. Many publications have reported the application of digital PCR measurement of gene copy number in FFPE tissues compared to FISH [11,12]. Here we analyzed 116 GC and 39 HCC PDX tumor models. The raw data are summarized in S2 Table. *MET* copy number analyzed by ddPCR is significantly elevated in the *MET* amplification group (mean, 15.4) compared to the *MET* non-amplification group (mean, 2.1) analyzed by FISH using a *t*-test with unequal variance (*P* = 0.044) (Fig 2). The linear association between the *MET* CN analyzed by ddPCR and the *MET/CEN-7* ratio analyzed by FISH was relatively strong, as evaluated by Pearson’s correlation (r = 0.782; *P* <0.001). Furthermore, using linear regression, the slope and intercept of the ratio of FISH to *MET* CN by ddPCR were significant (*P* < 0.001), with a value equal to 2.723 (standard errors of estimates = 0.176) and -0.803 (standard errors of estimates = 0.272), respectively (Fig 3). Generally, the results suggested that the *MET* copy number values obtained by ddPCR could distinguish between the *MET* non-amplification and amplification groups defined by FISH and that ddPCR can be used to measure the *MET* copy number in PDX models.

The concordance rate and Cohen's kappa coefficient were used to observe the consistency of gene amplification between ddPCR and FISH and evaluate whether there was a good cut-off value for defining the ddPCR-based gene amplification compared to the FISH ratio >2.0. Among the cut-off candidates, *MET* CN >5.5 by ddPCR had the highest concordance rate (98%) and Cohen's kappa coefficient (κ = 0.760) (95% CI, 0.498–1.000; *P* <0.001). Notably, among the 155 valid PDX samples, although most of samples had the consistency of *MET* gene amplification between ddPCR and FISH, only 3 PDX models (GAPF528, GAPF509, and GAPF012) that were positive for *MET* CN by ddPCR did not show a detectable FISH ratio >2.0 (Table 1).

## Discussion

Human genomes exhibit segmental copy number variation (CNV) at thousands of loci [13]. *MET* gene amplification has been described in gastric cancer (GC) and hepatocellular carcinoma (HCC), which results in dysregulation of MET signaling and is associated with clinical prognosis and poor outcome [6]. The aberrant MET signaling has been regarded as a robust target in the anti-cancer therapy. Thus, it is conceivable that a number of studies [7–9] have demonstrated *MET* gene amplification as a potential biomarker to estimate patients with cancers who will benefit from the treatment with selective MET inhibitors or antibodies. So far, there has been no standardized method to validate *MET* gene amplification or *MET* copy number.

SNP 6.0 and FISH are the methods that are conventionally used to evaluate *MET* copy number in cancer cell lines *in vitro* and tumor samples *in vivo*, respectively. However, a number of issues can complicate the detection of gene amplification. First, these methods are very costly and require advanced technical skills, and thus, informatics technicians or experienced pathologists must analyze the data and make subjective judgments. Second, *in vivo* tumor samples, such as FFPE tissues, are always tested by FISH and use long fragment probes, resulting in variations in the gene copy number detection depending on the probes targeting different gene loci. Thus, we sought to identify a method with simple technical requirements to more accurately detect the *MET* copy number.

In this study, we evaluated the feasibility of using a novel method of ddPCR to quantify the *MET* copy number or assess gene amplification from cancer samples consisting of cell lines or FFPE tumors. The results showed that ddPCR could determine the MET status in tumor samples. We found there was a positive correlation between the *MET* copy numbers detected by ddPCR and SNP 6.0 according to Pearson’s correlations (r = 0.867; *P* <0.001). Although microarray technologies are valuable approaches for CNV determination, they have limited dynamic range and are expensive for high-throughput screening in population studies [10]. Because of the limitations of SNP 6.0 approach in accurately measuring copy numbers greater than 4 and lack of sensitivity and resolution, ddPCR can be developed to measure high-copy CNV more accurately in a large numbers of samples, as described before [13]. Based on the assessment of *MET* amplification in 155 FFPE PDX tumors, ddPCR can reliably reflect the *MET* amplification status compared to FISH, with a 98% concordance rate. Moreover, there is significant difference of *MET* CNV detected by ddPCR between FISH-positive and FISH-negative groups, providing a highly significant proof of principle.

Notably, although in most of 155 FFPE PDX tumors, status of MET detected by ddPCR were comparable to those detected by FISH, three FFPE tumors whose *MET* copy number values were high (CN >5.5) in ddPCR did not show gene amplification by FISH (*MET/CEN-7* ratio >2.0). The reason for the discordance of the *MET* amplification between ddPCR and FISH may be the underestimation of *MET* amplification by FISH or overestimation of *MET* amplification by digital PCR. On the one hand, the presence of *MET* gene amplification could be masked because of characteristic of incomplete hybridization of long fragment probes used in FISH, resulting in non-specific identification of *MET* amplification. In particular, the variation in the *MET* amplification could be ascribed to the probe targeting different genomic loci detected by FISH. On the other hand, the fixation process used for the FFPE tissues could cause DNA fragmentation, damage or fusion, thus leading to a false-negative result. Additionally, considering that loss of chromosome frequently occurred in many cancers can result in genomic chromosomal instability [14–16], it implies that the loss of the reference gene loci in the tumor may cause a false-positive elevated ratio of *MET*:*AP3B1* in ddPCR, rather than high *MET* copy number.

Of note, *MET* CN of another PDX sample (GAPF101) is 0.00022 with no successful CN call by ddPCR, extremely lower than *MET/CEN-7* ratio of 1 by FISH. It implies that the deletion or damage of *MET* might occur in partial region of MET which were targeted by different probes employed in ddPCR and FISH, thus resulting in the difference of MET status between FISH and ddPCR. It will be intriguing to explore the reasons for the discordance in the future research.

Otherwise, the analysis of tumor samples by FISH requires complex experimental technology and may depend on the subjective judgment of pathologists. The results can vary between different pathologists based on their experiences. In this study, we show that ddPCR is a quantitative method that can absolutely quantify the *MET* copy number in cancer samples either *in vitro* or *in vivo*, without the need for standard curves or endogenous control.

Overall, these data suggest that ddPCR has the potential to detect the *MET* copy number in cancer cell lines or FFPE DNA and highly correlated with SNP 6.0 or FISH, respectively. *MET* gene amplification has been described to be associated with tumorigenesis and metastatic progression and is regarded as a biomarker to predict benefit from MET-targeted therapy in various clinical studies [7–9]. Our findings provide the insight that the ddPCR platform may be able to estimate the relationship between the MET status and patient prognosis in clinical studies and help to better predict and screen the patients in the response to MET-targeted therapy.

## Supporting Information

S1 Table Comparison of *MET* copy number detected by ddPCR versus by SNP 6.0. (PDF)

S2 Table Comparison of *MET* copy number detected by ddPCR versus by FISH. (PDF)
