# Supplementary material for: Detection of MET Gene Copy Number in Cancer Samples Using the Droplet Digital PCR Method
Source: PLoS One. 2016 Jan 14;11(1):e0146784. doi: 10.1371/journal.pone.0146784 (PMC4713204; doi:10.1371/journal.pone.0146784)
Supplement: S1 Table — (PDF) [file pone.0146784.s001.pdf]

S1 Table . Comparison of *MET* copy number detected by ddPCR versus by SNP 6.0

Cell line	Tissue	MET CN (ddPCR)	MET CN (SNP6.0)
Hs746T	GC	29	13
MKN45	GC	22.2	14
SNU-5	GC	15.6	8
SNU-620	GC	41.8	14
MKN1	GC	2.13	4
AZ521	GC	1.81	2
NUGC-4	GC	5.7	4
SNU-16	GC	2.49	3
BEL-7402	HCC	1.39	2
HCCC9810	HCC	5.64	5
Hep3B	HCC	2.99	3
HepG2	HCC	2.26	2
HLE	HCC	1.07	2
HLF	HCC	1.29	2
HuCCT1	HCC	1.65	3
HUH-1	HCC	2.52	3
HUH-28	HCC	1.39	2
HUH6_clone5	HCC	2.05	3
HUH-7	HCC	2.98	3
JHH-1	HCC	2.85	6
JHH-2	HCC	3.52	5
JHH-4	HCC	2.15	5
JHH-5	HCC	1.75	3
JHH-6	HCC	4.01	4
JHH-7	HCC	2.17	4
Li-7	HCC	1.95	3
NOZ	HCC	1.62	3
OCUG-1	HCC	5.52	8
OZ	HCC	2.63	3
PLC-PRF-5	HCC	3.56	4
QGY-7701	HCC	1.42	4
QGY-7703	HCC	1.53	3
SK-HEP-2	HCC	2.49	4
SMMC-7721	HCC	1.63	2
SNU-182	HCC	1.41	3
SNU-354	HCC	2.11	4
SNU-368	HCC	2.82	5
SNU-387	HCC	2.35	3
SNU-398	HCC	2.68	3
SNU-423	HCC	1.92	4
SNU-449	HCC	2.77	3
SNU-475	HCC	2.11	3
SNU-739	HCC	2.11	4
SNU-761	HCC	2.36	2
SNU-878	HCC	2.31	3
SNU-886	HCC	2.68	4
