# Supplementary material for: Detection of MET Gene Copy Number in Cancer Samples Using the Droplet Digital PCR Method
Source: PLoS One. 2016 Jan 14;11(1):e0146784. doi: 10.1371/journal.pone.0146784 (PMC4713204; doi:10.1371/journal.pone.0146784)
Supplement: S2 Table — (PDF) [file pone.0146784.s002.pdf]

S2 Table. Comparison of *MET* copy number detected by ddPCR versus by FISH

Code	Model	Tissue	MET CN (ddPCR)	*MET/CEP7 ratio*	Positive/Negative (P/N)
1	GAPF438	GC	1.89	1.13	N
2	GAPF344	GC	2.2	1.02	N
3	GAPF528	GC	17.01	1.13	N
4	GAPF308	GC	1.61	1.02	N
5	GAPF559	GC	5.92	6.79	P
6	GAPF381	GC	12.98	5.46	P
7	GAPF478	GC	1.77	1	N
8	GAPF847	GC	1.63	1.07	N
9	GAPF510	GC	1.184	1.02	N
10	GAPF854	GC	1.97	0.95	N
11	GAPF451	GC	1.89	0.97	N
12	GAPF403	GC	1.35	0.95	N
13	GAPF493	GC	0.334	0.98	N
14	GAPF375	GC	2.59	1.09	N
15	GAPF916	GC	1.501	1.07	N
16	GAPF304	GC	1.68	0.8	N
17	GAPF339	GC	2.24	1	N
18	GAPF509	GC	11.1	0.98	N
19	GAPF570	GC	2.37	1.1	N
20	GAPF843	GC	1.352	1.11	N
21	GAPF439	GC	6.82	4.3	P
22	GAPF526	GC	3.34	1.24	N
23	GAPF811	GC	1.331	0.97	N
24	GAPF405	GC	2.78	1.07	N
25	GAPF436	GC	1.63	1.05	N
26	GAPF374	GC	3.05	1.14	N
27	GAPF462	GC	1.249	1.03	N
28	GAPF480	GC	2	1.19	N
29	GAPF516	GC	3.43	1.05	N
30	GAPF563	GC	1.246	0.97	N
31	GAPF878	GC	2.57	1.06	N
32	GAPF525	GC	3.35	1.24	N
33	GAPF534	GC	2.05	1.11	N
34	GAPF599	GC	1.6	1.02	N
35	GAPF461	GC	1.359	0.97	N
36	GAPF535	GC	3.1	1.07	N
37	GAPF383	GC	21.2	6.13	P
38	GAPF479	GC	2.09	1.17	N
39	GAPF907	GC	0.805	1.1	N

Code	Model	Tissue	MET CN (ddPCR)	*MET/CEP7* ratio	Positive/Negative (P/N)
40	GAPF455	GC	1.4	1.07	N
41	GAPF613	GC	2.26	1.04	N
42	GAPF591	GC	1.092	0.88	N
43	GAPF536	GC	2.88	1.31	N
44	GAPF553	GC	1.63	1.08	N
45	GAPF600	GC	1.74	0.95	N
46	GAPF547	GC	2.63	1.32	N
47	GAPF804	GC	2.27	1	N
48	GAPF846	GC	1.57	1.18	N
49	GAPF508	GC	1.88	0.95	N
50	GAPF562	GC	1.54	1.03	N
51	GAPF494	GC	1.28	0.8	N
52	GAPF512	GC	1.28	0.89	N
53	GAPF538	GC	2.88	0.98	N
54	GAPF801	GC	0.841	0.97	N
55	GAPF879	GC	2.23	1.06	N
56	GAPF580	GC	2.48	1.03	N
57	LIPF042	HCC	1.56	1.05	N
58	LIPF247	HCC	1.6	1.28	N
59	LIPF252	HCC	1.76	1.06	N
60	LIPF255	HCC	1.89	1.42	N
61	LIPF256	HCC	2.7	1.06	N
62	LIPF261	HCC	4.11	1.18	N
63	LIPF262	HCC	2.01	1.02	N
64	LIPF264	HCC	1.177	1.33	N
65	LIPF270	HCC	1.69	1.14	N
66	LIPF274	HCC	0.929	0.9	N
67	LIPF277	HCC	2.8	1.73	N
68	LIPF282	HCC	0.83	0.62	N
69	GAPF057	GC	0.954	0.88	N
70	GAPF069	GC	1.78	1.03	N
71	GAPF112	GC	1.84	1.13	N
72	GAPF115	GC	1.54	0.97	N
73	GAPF132	GC	1.93	1.02	N
74	GAPF147	GC	1.51	1.1	N
75	GAPF158	GC	1.445	1	N
76	GAPF161	GC	2.03	1.09	N
77	GAPF162	GC	0.552	0.68	N
78	GAPF170	GC	0.205	1.11	N

Continued S2 Table. Comparison of *MET* copy number detected by ddPCR versus by FISH

Code	Model	Tissue	MET CN (ddPCR)	*MET/CEP7 ratio*	Positive/Negative (P/N)
79	GAPF187	GC	2.61	0.98	N
80	GAPF188	GC	1.93	1.09	N
81	GAPF190	GC	2	1.08	N
82	GAPF195	GC	0.864	0.89	N
83	GAPF209	GC	0.951	1.03	N
84	GAPF210	GC	2.66	1.3	N
85	GAPF220	GC	2.15	1.28	N
86	GAPF221	GC	2.54	0.96	N
87	GAPF101	GC	0.00022 (no call)	1	N
88	GAPF238	GC	3.22	1.45	N
89	GAPF241	GC	1.84	0.83	N
90	GAPF118	GC	1.65	1.05	N
91	GAPF250	GC	1.525	1.02	N
92	LIPF335	HCC	1.8	1.3	N
93	LIPF021	HCC	1.86	1.32	N
94	LIPF025	HCC	3.09	1.05	N
95	LIPF028	HCC	1.41	1.22	N
96	LIPF037	HCC	2.13	1.14	N
97	LIPF039	HCC	3.35	1.02	N
98	LIPF048	HCC	1.77	1.19	N
99	LIPF067	HCC	5.06	1.33	N
100	LIPF086	HCC	1.67	1.13	N
101	LIPF088	HCC	1.84	0.8	N
102	LIPF099	HCC	1.53	1.06	N
103	LIPF134	HCC	1.46	0.97	N
104	LIPF139	HCC	2.82	0.57	N
105	LIPF143	HCC	1.017	0.92	N
106	LIPF159	HCC	1.77	1.17	N
107	LIPF166	HCC	1.89	1.09	N
108	LIPF177	HCC	1.25	1.09	N
109	LIPF181	HCC	0.623	0.9	N
110	LIPF185	HCC	2.88	0.97	N
111	LIPF191	HCC	1.76	0.93	N
112	LIPF210	HCC	30.2	8.43	P
113	LIPF233	HCC	1.95	1.06	N
114	LIPF236	HCC	1.65	1	N
115	LIPF239	HCC	0.943	0.72	N
116	LIPF245	HCC	1.63	0.96	N
117	LIPF250	HCC	1.297	1.02	N

Code	Model	Tissue	MET CN (ddPCR)	*MET/CEP7 ratio*	Positive/Negative (P/N)
118	LIPF280	HCC	1.73	1.09	N
119	GAPF006	GC	1.416	1.06	N
120	GAPF008	GC	2.16	1.08	N
121	GAPF012	GC	7.54	1.11	N
122	GAPF015	GC	1.92	1.06	N
123	GAPF016	GC	3.23	1.19	N
124	GAPF023	GC	3.22	1.14	N
125	GAPF027	GC	2.87	1.07	N
126	GAPF038	GC	1.7	1	N
127	GAPF039	GC	2.35	1.17	N
128	GAPF041	GC	1.258	1.03	N
129	GAPF047	GC	1.034	1.06	N
130	GAPF048	GC	3.4	1.07	N
131	GAPF054	GC	1.617	1	N
132	GAPF077	GC	2.74	1.02	N
133	GAPF084	GC	0.264	0.98	N
134	GAPF089	GC	1.81	0.84	N
135	GAPF094	GC	1.379	1.06	N
136	GAPF097	GC	1.31	1	N
137	GAPF108	GC	2.12	1.06	N
138	GAPF113	GC	1.105	0.99	N
139	GAPF114	GC	5.35	1.17	N
140	GAPF129	GC	3.2	1.08	N
141	GAPF140	GC	1.146	1.09	N
142	GAPF155	GC	1.76	1.06	N
143	GAPF157	GC	1.144	1.05	N
144	GAPF159	GC	1.233	0.72	N
145	GAPF302	GC	1.57	1.03	N
146	GAPF313	GC	1.61	1.13	N
147	GAPF314	GC	1.93	1.19	N
148	GAPF329	GC	1.02	0.96	N
149	GAPF346	GC	2.34	0.98	N
150	GAPF378	GC	1.54	1.03	N
151	GAPF380	GC	1.45	1.06	N
152	GAPF387	GC	2.9	1.07	N
153	GAPF406	GC	1.006	0.92	N
154	GAPF407	GC	1.32	0.98	N
155	GAPF414	GC	1.91	0.96	N
